# The Brain–Heart Axis: An Umbrella Review on Impact of Psychiatric Disease on Incidence, Management, and Outlook of Cardiovascular Disease

**DOI:** 10.3390/life14080919

**Published:** 2024-07-23

**Authors:** Marianna Mazza, Giuseppe Biondi-Zoccai, Francesco Maria Lisci, Caterina Brisi, Greta Sfratta, Sara Rossi, Gianandrea Traversi, Eleonora Gaetani, Roberto Pola, Sofia Morini, Enrico Romagnoli, Benedetta Simeoni, Marcello Covino, Giuseppe Marano

**Affiliations:** 1 Unit of Psychiatry, Fondazione Policlinico Universitario A. Gemelli IRCCS, 00168 Rome, Italy; 2 Department of Neurosciences, Università Cattolica del Sacro Cuore, 00168 Rome, Italy; 3 Department of Medical-Surgical Sciences and Biotechnologies, Sapienza University of Rome, 04100 Latina, Italy; 4 Maria Cecilia Hospital, GVM Care & Research, 48033 Cotignola, Italy; 5 Unit of Medical Genetics, Department of Laboratory Medicine, Ospedale Isola Tiberina-Gemelli Isola, 00186 Rome, Italy; gianandrea.traversi@gmail.com; 6 Department of Translational Medicine and Surgery, Fondazione Policlinico Universitario A. Gemelli IRCCS, Università Cattolica del Sacro Cuore, 00168 Rome, Italy; 7 Unit of Internal Medicine, Cristo Re Hospital, 00167 Rome, Italy; 8 Section of Internal Medicine and Thromboembolic Diseases, Department of Internal Medicine, Fondazione Policlinico Universitario A. Gemelli IRCCS, Università Cattolica del Sacro Cuore, 00168 Rome, Italy; 9 Department of Cardiovascular Sciences, Fondazione Policlinico Universitario A. Gemelli IRCCS, 00168 Rome, Italy; 10 Emergency Department, Fondazione Policlinico Universitario A. Gemelli IRCCS, 00168 Rome, Italy

**Keywords:** anxiety, bipolar disorder, cardiovascular disease, depression, heart failure, myocardial infarction, schizophrenia

## Abstract

Psychiatric conditions, such as depression, anxiety, bipolar disorder, and schizophrenia, are increasingly recognized as significant risk factors for cardiovascular disease (CVD). This review systematically analyzes evidence from various databases to provide a comprehensive understanding of the impact of psychiatric illnesses on the incidence, management, and prognosis of CVD. Key findings suggest a bidirectional relationship between psychiatric disorders and CVD, indicating that mental health conditions can predispose individuals to CVD, while CVD can exacerbate or trigger psychiatric symptoms. The review explores the underlying mechanisms of these associations, including behavioral factors, stress responses, and medication side effects. It also examines the challenges in managing CVD patients with comorbid psychiatric conditions, emphasizing the importance for integrated care approaches. This review underscores the necessity of considering mental health as an integral component of cardiovascular care and calls for further research to develop tailored management strategies for these complex conditions, ultimately aiming to improve patient outcomes and quality of life. This comprehensive analysis provides valuable insights for future investigations and guides clinicians in optimizing care for patients with both psychiatric and cardiovascular conditions.

## 1. Introduction

Cardiovascular diseases (CVDs) are the main cause of death in the U.S. and worldwide, with coronary heart disease (CHD) being the most common type of CVD. Angina and myocardial infarction (MI) are two manifestations of CHD, and MI is the principal cause of death from CHD [1]. The main pathophysiological mechanism of CHD is the accumulation of a waxy substance, known as atheromatous plaque (or atheroma), in the heart’s arteries, which compromises coronary circulation. The presence of the plaque reduces blood flow and increases the probability of a clot forming, leading to artery blockage and resulting in myocardial infarction (MI). The development of CHD is a complex process influenced by various factors, including genetic predisposition, family history of CHD, smoking, sedentary lifestyle, stress, older age, male gender, diabetes, obesity, and dyslipidemia [2].

In this complex scenario, recent evidence has showed a strong correlation between CVD and mental illnesses, suggesting the need for additional research. Mental illness and psychological pain are significant contributors to the global burden of diseases, with over 300 million people worldwide suffering from depression, which is expected to become the leading cause of disability in 2030. According to Organization for Economic Co-operation and Development (OECD) data, at least 84 million people in the EU suffered from mental illnesses before the COVID-19 pandemic, with 1 in 6 people in the EU (17.3%) affected. Anxiety and depression were the most common mental illnesses, followed by disorders related to alcohol and drug use. A total of 25 million individuals experienced anxiety disorders (5.4% of the total EU population), 21 million people suffered from depressive disorders (4.5% of the total EU population), and 11 million individuals were affected by related disorders to the use of alcohol and drugs (2.4%) [3]. According to WHO estimates, this situation has worsened on a worldwide level. A Eurobarometer survey conducted in June 2023 revealed that almost 1 in 2 people (46% of the total EU population) had experienced emotional or psychosocial distress, such as symptoms of depression or anxiety, in the previous 12 months [4].

Given the distribution and prevalence of psychiatric disorders in the global population, their higher prevalence in CHD patients, and mental disease being a risk factor for CHD, recent investigations have placed greater emphasis on exploring the correlation between these two health conditions [2].

For example, acute coronary syndromes (ACS) and major depressive disorders are recognized as important factors in the development and worsening of each other’s clinical course [5]. Major Depressive Disorder (MDD) is one of the most common comorbidities among individuals with medical diseases, with a point prevalence of over 10% or even 20%, not to mention patients who have subclinical depressive symptoms, which may be even more. Treating depression, as well as other psychiatric diseases, alongside medical condition should be considered as a primary intervention, because of the reduced quality of life and poorer prognosis that result from the under-treatment of mental illnesses [6].

Indeed, clinical depression among patients with myocardial infarction has been associated with reduced adherence to prescribed lifestyle changes and decreased compliance with medical treatments [7]. Post-stroke depression can hide or delay patients’ progress in rehabilitation, extending hospital admissions, thereby adding to the economic burden of the disease. Despite this, depression is still under-recognized, undertreated, and often considered as a consequence of cardiovascular diseases, rather than a risk factor.

In 2008, the American Heart Association issued an advisory for screening for depression in patients with coronary heart disease, but early reports on proposed screening protocols showed poor implementation [8]. Nonetheless, identifying signs of depression in post-acute myocardial infarction (AMI) patients offers an opportunity to improve overall medical care and address a significant health concern. Studies such as SADHEART and ENRICHD suggest that selective serotonin-reuptake inhibitors are not only safe but also effective in treating depression in AMI patients [9,10]. In addition to drug treatment, patients post-ACS require cardiological rehabilitation in order to enhance physical performance and reduce the risk of future adverse cardiological events [11].

Schizophrenia is another psychiatric disorder associated with worsened outcomes when comorbid with acute coronary syndrome [12]. It is a psychiatric condition characterized by delusions, disorganized speech, hallucinations, and impaired executive functioning, affecting approximately 1% of the world’s population and ranking as one of the top 10 causes of global disability [13]. Individuals suffering from schizophrenia are at a higher risk of developing cardiovascular diseases (CVD) compared to those without mental health issues. This heightened risk is attributed to a higher prevalence of CVD risk factors such as obesity, metabolic disorders, diabetes, and smoking [11,13].

Patients affected by schizophrenia and acute myocardial infarction are at a higher risk of all-cause mortality compared to those without schizophrenia. Additionally, it has been observed that schizophrenic patients with AMI may have lower rates of revascularization procedures compared to those without schizophrenia. This suggests that individuals suffering from schizophrenia and AMI could face limited access to medical care, particularly procedures like percutaneous coronary intervention (PCI) or coronary artery bypass graft (CABG), which are recognized as the most effective methods for coronary revascularization in cases of blocked coronary arteries [14].

A consistent trend of higher mortality rates has been described in association with First-Generation Antipsychotics (FGAs) compared to Second-Generation Antipsychotics (SGAs). The differences in mortality rates may be partly attributed to occurrences such as stroke, hip fracture, acute myocardial infarction, and ventricular arrhythmia [15]. It is also well known that corrected QT interval (QTc) prolongation is one of the possible complications in patients with schizophrenia who are taking antipsychotics, which can lead to malignant cardiac arrhythmia [16]. Other drugs that can increase cardiovascular adverse events in older people are stimulants, which are commonly prescribed in the treatment of attention deficit hyperactivity disorder (ADHD). The mechanisms through which they contribute to this are increases in heart rate and blood pressure, vasospasms caused by circulating catecholamines, and by prolonging the QT interval [17].

Psychopathological mechanisms are related to the presence of cardiac risk factors and the development of cardiovascular diseases. The objective of this article is to explore the correlations between mental disorders and CHD with the aim of preventing and treating both effectively.

## 2. Materials and Methods

This work was designed as an umbrella review (i.e., overview of systematic reviews) following established recommendations for evidence synthesis [18]. Specifically, we looked for systematic reviews on the interplay between psychiatric and cardiovascular disease limiting our search to PubMed, using the following string: https://pubmed.ncbi.nlm.nih.gov/?term=%28psychiat*+OR+depress*+OR+schizophr*+OR+%28mood+AND+disord*%29%29+AND+myocardial+AND+infarction&filter=pubt.systematicreview&sort=date&size=200 (accessed on 1 September 2023). We restricted our search to studies published up to 1 September 2023. Citations were initially screened at the title/abstract level. Subsequently, full texts were obtained for potentially relevant articles. Salient features of the included reviews and corresponding original studies were extracted when applicable. Review quality was appraised using the Oxman–Guyatt index [18]. Quantitative data were summarized using the median (minimum–maximum) or count (%), as appropriate. Notably, given the design features of our umbrella review (i.e., the focus on aggregate data only), no study- or patient-level analysis was conducted.

## 3. Results

From a total of 156 screened titles, we eventually included 49 systematic reviews (Table 1 and Table 2). They were published between 2004 and 2023, including a total of 1221 studies (range: 5; 122) and a total of 48,842,606 patients (range: 280; 13,115,911). A meta-analysis was conducted in 22 (46%) of systematic reviews.

The main findings of these overviews are detailed in Table 3, and their main limitations in Table S1. First, there was a significant relationship between psychiatric disorders such as depression, anxiety, and schizophrenia and cardiovascular diseases, showing substantial patterns of comorbidity [19,20,21,22,23,24,25,26,27,28,29,30]. Most systematic reviews indicated that patients with depressive and anxiety disorders were at increased risk of myocardial infarction and stroke [19,22,26,31,32,33,34,35,36]. Similarly, schizophrenia was associated with higher post-acute coronary syndrome mortality and complications [28,35].

The effect of psychiatric medications on cardiovascular health varied significantly. For example, while antidepressants are generally considered safe, their impact on cardiovascular outcomes has shown mixed results [37,38,39,40,41]. Some studies suggested that there is no significant association between ADHD medications and cardiovascular events, while others have reported potential cardiovascular risks associated with the use of antipsychotic [23]. Psychological interventions, such as cognitive-behavioral therapy and psychoeducation, have shown promise in reducing symptoms of depression and potentially improving cardiovascular health [29,36,42,43,44,45,46,47,48,49,50,51,52,53,54,55,56,57,58,59,60,61,62,63,64,65,66,67]. Pharmacological treatments, such as antidepressants in post-acute coronary syndrome patients, have been found to reduce depressive symptoms without significantly impacting mortality but there have been favorable trends for reductions in hospital readmissions [31,32,34,37]. Furthermore, psychiatric conditions have been shown to meaningfully affect mortality and treatment outcomes in cardiovascular patients. Studies consistently have demonstrated that patients with severe mental illnesses received less aggressive treatment following cardiac events and had higher mortality rates as a result [66].

Socio-demographic factors such as marital status and a history of child abuse have clearly been shown to affect cardiovascular outcomes in psychiatric patients [21]. Specifically, married or partnered individuals reported better functional outcomes post-myocardial infarction, and a history of child abuse is associated with an increased risk of coronary heart disease in adulthood [20]. Therefore, it is important to identify and manage risk factors for cardiovascular disease in psychiatric patients to prevent adverse outcomes. Depression is a common issue following acute coronary syndrome and often remains undertreated, leading to higher morbidity and increased hospital readmissions. The cardiac rehabilitation and proactive management of depressive symptoms have clearly demonstrated to play significant roles in reducing these risks and improving patient outcomes. Psychosocial stressors, emotional and cognitive reactions to life events, and overall quality of life significantly impacted the cardiovascular health of individuals with psychiatric disorders. For instance, major cardiac event survivors appeared at significant risk of developing post-traumatic stress symptoms, which can affect their long-term recovery and quality of life [30,36].

**Table 1:** Description of included studies with an explicit cardiovascular focus.

First Author	Journal	Year	PubMed ID	Studies	Sample Size	Meta-Analysis	Patient Selection Criteria	Methodological Selection Criteria	Review Features
**Zhu C. [20]**	*Eur Heart J Open*	2023	36942107	34	16,712	Yes	Patients with MI	Clinical (observational or RCTs) studies investigating the association between marital/partner status and MI	Meta-analysis
**Aw P.Y. [22]**	*J Psychosom Res*	2023	36610338	48	57,342	Yes	Patients with anxiety/depression and MI/stroke	Clinical (observational or RCTs) studies evaluating the co-prevalence of anxiety/depression and MI/stroke	Meta-analysis
**Chan J.K.N. [24]**	*Schizophr Bull*	2022	35786737	22	12,235,501	Yes	Patients with recent ACS	Clinical (observational or RCTs) studies investigating the association between psychiatric disease and outcomes after ACS	Meta-analysis
**Jacquet-Smailovic M. [25]**	*Journal of Traumatic Stress*	2022	34715167	39	10,312	Yes	Patients with MI screened for ASD, PTSD, and/or PTSS	Clinical (observational or RCTs) studies investigating the association between anxiety and depression, personality traits, emotional and/or cognitive reactions, quality of life, social support, and potentially stressful events	Meta-analysis
**Khan Z. [26]**	*Cureus*	2021	35141096	7	4776	Yes	Patients with recent ACS	Clinical (observational or RCTs) studies investigating the association between depression and outcomes after ACS	Meta-analysis
**Tully P.J. [27]**	*Cochrane Database of Systematic Reviews*	2021	34910821	37	9225	Yes	Individuals with CAD and depression	Clinical (observational or RCTs) studies investigating the association between the effect of psychological and pharmacological interventions and depression in CAD patients	Meta-analysis
**Hannoodee H. [28]**	*Cureus*	2021	34540400	14	5,270,554	No	ACS patients with or without a concurrent diagnosis of schizophrenia	Clinical (observational or RCTs) studies investigating the association between schizophrenia and mortality or morbidity outcomes following an initial event of ACS	Systematic review
**Sancassiani F. [29]**	*Journal of Clinical Medicine*	2021	34501261	36	10,389	No	People with AMI	Clinical (observational or RCTs) studies investigating the effectiveness of psychological and educational interventions compared to usual care	Systematic review
**Cojocariu S.A. [30]**	*Journal of Personalized Medicine*	2021	34063747	11	3090	No	Patients with acute coronary syndrome	Clinical (observational or RCTs) studies investigating the association between post-traumatic stress disorder (PTSD) and ischemic heart disease	Systematic review
**Jacquet-Smailovic M. [31]**	*Journal of Traumatic Stress*	2021	33007150	39	10,312	No	Patients with MI	Clinical (observational or RCTs) studies investigating the association between the antidepressant therapy and mortality/cardiovascular outcomes in patients with ACS	Systematic review
**Fernandes N. [32]**	*Clinical Research in Cardiology*	2021	32617669	8	1171	Yes	Patients with depression after ACS and CAD	Clinical (observational or RCTs) studies investigating the association between the effects of SSRIs and cardiovascular events in depressed CAD patients	Meta-analysis
**Sweda R.** **[33]**	*ESC Heart Failure*	2020	32935927	10	1935	Yes	Patients with ACS and concomitant depression	Clinical (observational or RCTs) studies investigating the association between the effect of antidepressant therapy and cardiovascular outcomes in patients with ACS	Meta-analysis
**Shao M. [35]**	*Progress in Neuropsychopharmacology & Biological Psychiatry*	2020	31954758	6	3,260,754	Yes	AMI patients with and without schizophrenia	Clinical (observational or RCTs) studies investigating the revascularization rate in schizophrenic patients after AMI	Meta-analysis
**Zheng X.** **[39]**	*Heart & Lung*	2019	30366575	20	1828	yes	MI patients with anxiety and/or depression	Clinical (observational or RCTs) studies investigating the association between exercise-based CR treatments and anxiety and depression symptoms in MI patients	Meta-analysis
**Ladwig S.** **[41]**	*Psychosomatic Medicine*	2018	30113911	49	19,705	yes	Patients with a clinical diagnosis of stroke or MI	Clinical (observational or RCTs) studies investigating the association between treatment rates and the application of guidelines in stroke and MI	Meta-analysis
**Richards S.H. [43]**	*European Journal of Preventive Cardiology*	2018	29212370	35	10,703	Yes	Patients following MI or revascularization or with a diagnosis of angina pectoris or CHD defined by angiography	Clinical (observational or RCTs) studies investigating the association between psychological intervention and mortality, cardiovascular morbidity, and psychological outcomes	Meta-analysis
**Richards S.H. [44]**	*Cochrane Library*	2017	28452408	35	10,703	no	Coronary heart disease (CHD) patients	Clinical (observational or RCTs) studies investigating the association between psychological interventions compared to usual care in adults with a specific diagnosis of CHD	Systematic review
**Yu Z.H. [45]**	*Br J Clin Pharmacol.*	2016	27198162	9	351,516	yes	MI incidence among patients receiving antipsychotics vs. no treatment	Clinical (observational or RCTs) studies investigating the association between AP and risk of MI	Meta-analysis
**Ski C.F. [46]**	*European Journal of Cardiovascular Nursing*	2016	26475227	5	3192	no	People with coronary heart disease and depression	Clinical (observational or RCTs) studies investigating the association between psychosocial interventions and depressive symptoms in patients with CHD and depression	Systematic review
**Doyle F. [48]**	*Psychosomatic Medicine*	2015	25886829	30	10,175	no	Patients post-MI	Clinical (observational or RCTs) studies investigating sex differences in depression andprognosis post-MI	Meta-analysis
**Janzon E. [49]**	*Scandinavian Journal of Psychology*	2015	25756318	10	2478	no	Patients who have suffered a cardiac event	Clinical (observational or RCTs) studies investigating whether physical activity can be a tool to reduce depression in patients who suffered a cardiac event	Systematic review
**Whalley B.** **[52]**	*International Journal of Behavioral Medicine*	2014	23179678	24	9296	no	Patients with CHD	Clinical (observational or RCTs) studies investigating the effects of psychological interventions in CHD patients	Meta-analysis
**Bradt J.** **[53]**	*Cochrane Database*	2013	24374731	26	1369	no	People with CHD	Clinical (observational or RCTs) studies investigating the effects of music interventions with standard care versus standard care alone on psychological and physiological responses in persons with CHD	Systematic review
**Foxwell R.** **[55]**	*J Psychosom Res*	2013	23972409	21	8452	no	Patients with CHD	Clinical (observational or RCTs) studies investigating the association between physical activity and depression in patients who have suffered a cardiac event	Systematic review
**Thombs B.D. [56]**	*PLoS One*	2013	23308116	18	6857	no	Patients with CHD	Clinical (observational or RCTs) studies investigating the effectiveness of depression screening in CHD	Systematic review
**Whalley B.** **[59]**	*Cochrane Database Syst Rev*	2011	21833943	24	9296	no	Patients with CHD	Clinical (observational or RCTs) studies investigating the effects of psychological interventions in patients with CHD	Systematic review
**Zuidersma M.** **[60]**	*Psychotherapy and Psychosomatics*	2011	21502770	6	3206	no	Patients with ACS and depression	Clinical (observational or RCTs) studies investigating the association between onset and recurrence of depression in ACS patients and cardiovascular prognosis	Systematic review
**Thombs B.D. [61]**	*JAMA*	2008	19001627	11	4381	no	Patients with CVD	Clinical (observational or RCTs) studies investigating the potential benefits of depression screening in patients with CVD	Systematic review
**Thombs B.D. [62]**	*Psychosomatics*	2007	17478586	7	3756	no	Patients after acute MI	Clinical (observational or RCTs) studies investigating the efficacy of depression screening in patients after AMI	Systematic review
**Van der Kooy K. [63]**	*Int J Geriatr Psychiatry*	2007	17236251	28	80,000	yes	People with depression and risk for CVD	Clinical (observational or RCTs) studies reporting depression at baseline and CVD outcomes at follow-up	Meta-analysis and meta-regression
**Frasure-Smith N. [65]**	*Psychosom Med*	2005	15953794	32	87,033	no	People with depression and risk for CVD	Clinical (observational or RCTs) studies investigating the association between depressive symptoms with cardiac disease outcomes	Systematic review
**Sørensenf C. [66]**	*Psychother Psychosom*	2005	15741756	31	33,913	no	Patients with MI	Clinical (observational or RCTs) studies assessing the methodological quality and investigating whether depression leads to an increased post-MI mortality	Systematic review
**Rees K. [67]**	*Cochrane Database Syst Rev*	2004	15106183	36	12,841	no	Patients with CHD	RCTs investigating the effectiveness of psychological interventions in patients with CHD	Systematic review

MI: myocardial infarction; CHD: coronary heart disease; CVD: cardiovascular disease; ACS: acute coronary syndromes; CAD: coronary artery disease; ASD: acute stress disorder; PTSD: post-traumatic stress disorder; PTSS: potentially stressful events; SSRIs: selective serotonin reuptake inhibitors; AMI: acute myocardial infarction; AP: antipsychotics; CR: cardiac rehabilitation; SGAs: second-generation antipsychotics.

**Table 2:** Description of included studies without an explicit cardiovascular focus.

First Author	Journal	Year	PubMed ID	Studies	Sample Size	Meta-Analysis	Patient Selection Criteria	Methodological Selection Criteria	Review Features
**Köhler-Forsberg O. [19]**	*JAMA Psychiatry*	2023	37672261	52	24,006	Yes	Patients with diagnosed medical disease and randomized to receive AD for depression	Systematic reviews of RCTs for treatment of comorbid depression in medical diseases	Umbrella review
**Chen Y. [21]**	*Am J Prev Med*	2023	36878413	10	343,371	Yes	Adults with or without any type of child abuse before age 18 years	Clinical (observational or RCTs) studies investigating the association between child abuse and CHD	Meta-analysis
**Zhang L. [23]**	*JAMA Netw Open*	2022	36416824	19	3,931,532	Yes	Individuals receiving ADHD medications	Clinical (observational or RCTs) studies investigating the association between ADHD medications and CVD	Meta-analysis
**Park K.** **[34]**	*Journal of Psychiatric Research*	2020	32135389	17	5452	Yes	Patients taking duloxetine for mood disorders or for controlling pain	Clinical (observational or RCTs) studies investigating the association between duloxetine and CAEs	Meta-analysis
**Haerizadeh M. [36]**	*Journal of Psychosomatic Research*	2020	31884302	6	280	no	Patients with PTSD induced by medical events	Clinical (observational or RCTs) studies investigating the optimal treatment of PTSD symptoms after medical events	Systematic review
**Papola D. [37]**	*Acta Psychiatrica Scandinavica*	2020	31260664	68	399,868	no	Patients taking AP	Clinical (observational or RCTs) studies investigating the association between hip fracture, thromboembolism, stroke, MI, pneumonia, sudden cardiac death, and exposure to antipsychotics	Umbrella review
**Zivkovic S.** **[38]**	*BMC Psychiatry*	2019	31221107	29	2,957,783	yes	Patients taking AP	Clinical (observational or RCTs) studies investigating the association between AP drug use and stroke or MI risk	Meta-analysis
**Benjenk I.** **[40]**	*Journal of Hospital Management and Health Policy*	2018	30283917	13	474,981	no	Patients initially hospitalized for various medical diseases	Clinical (observational or RCTs) studies investigating the association between interventions deigned to assess or treat mental health symptoms and risk of readmission following hospitalization for physical health conditions	Systematic review
**Eurelings L.S. [42]**	*Clinical Epidemiology*	2018	29670402	21	47,625	yes	Older people with apathy symptoms and/or depressive symptoms	Clinical (observational or RCTs) studies investigating the association between apathy and depressive symptoms in older people and future CVD, stroke, mortality	Meta-analysis
**Tully P.J. [47]**	*Psychological Medicine*	2015	26027689	12	1,131,612	no	The population of interest was people with PD at baseline but without verified or known CHD at this time from the general, cardiology or psychiatric population (inpatients and outpatients)	Clinical (observational or RCTs) studies investigating the association between PD, related syndromes, and incident CHD	Systematic review
**Jackson J.W.** **[50]**	*PLoS One*	2014	25140533	20	798,052	yes	Older adults using FGAs or SGAs	Clinical (observational or RCTs) studies investigating mortality and medical event risk between FGAs and SGAs in older adults	Meta-analysis
**Prieto M.L.** **[51]**	*Acta Psychiatr Scand*	2014	24850482	5	13,115,911	yes	Patients with BD	Clinical (observational or RCTs) studies investigating the risk of MI and stroke in people with bipolar disorder	Meta-analysis
**Health Quality Ontario** **[54]**	*Ont Health Technol Assess Ser*	2013	24133570	9	8042	no	Patients with chronic diseases	Clinical (observational or RCTs) studies investigating the effectiveness of screening for depression and/or anxiety in adults with chronic diseases	Systematic review
**Westover A.N. [57]**	*BMC Cardiovasc Disord*	2012	22682429	10	4,017,420	no	People using stimulants	Clinical (observational or RCTs) studies investigating the association between prescription stimulant use and adverse cardiovascular outcomes	Systematic review
**Prochaska J.J. [58]**	*BMJ*	2012	22563098	22	9232	no	Current tobacco users of adult age	Clinical (observational or RCTs) studies investigating the association between serious CAEs and varenicline use	Meta-analysis
**Swenson J.R. [64]**	*Can J Psychiatry*	2006	17249635	122	13,828	no	High-risk patients	RCTs investigating whether SSRIs are associated with an increased or decreased risk of CAEs	Systematic review

AD: antidepressants; RCTs: randomized controlled trials; MI: myocardial infarction; CHD: coronary heart disease; ADHD: attention deficit hyperactivity disorder; CVD: cardiovascular disease; ACS: acute coronary syndromes; CAD: coronary artery disease; CAEs: cardiovascular adverse events; ASD: acute stress disorder; PTSD: post-traumatic stress disorder; PTSS: potentially stressful events; SSRIs: selective serotonin reuptake inhibitors; AMI: acute myocardial infarction; AP: antipsychotics; CR: cardiac rehabilitation; PD: panic disorder; FGAs: first-generation antipsychotics; SGAs: second-generation antipsychotics.

**Table 3:** Outcomes, main findings, and limitations of included studies.

First Author	Year	Outcomes	Main Findings	Limitations
**Köhler-Forsberg O. [19]**	2023	Efficacy and safety of AD for treatment or prevention of comorbid depression in any medical disease, AD acceptability, and AD tolerability	ADs are effective and safe in treating and preventing depression in patients with comorbid medical disease	Few included RCTs
**Zhu C. [20]**	2023	Association between marital/partner status and patient-reported outcome measures following MI	Functional outcomes after MI are better for married/partnered individuals	Definitions for outcomes and assessment of marital/partner status differed across studies
**Chen Y. [21]**	2023	Association between child abuse with adult CHD risk	Child abuse is associated with an increased risk of adult CHD	Few prospective studies, prevalence of child abuse could have been underestimated
**Aw P.Y. [22]**	2023	Incidence and prevalence of anxiety/depression and MI/stroke	There is substantial comorbidity pattern between anxiety/depression and MI/stroke	Short follow-up, substantial heterogeneity
**Zhang L. [23]**	2022	Association between ADHD medications with the risk of a broad range of CVDs	There was no statistically significant association between ADHD medications and the risk of cardiovascular events among people of all ages	Heterogeneity due to a lack of data, investigation of the dose–response association was not possible
**Chan J.K.N. [24]**	2022	People with SMI may experience excess mortality and inequitable treatment following ACS	SMI is associated with increased post-ACS mortality and undertreatment	High heterogeneity: pooled analyses for BD, specific categories of MACEs, and individual cardioprotective drug classes were conducted on a limited number of studies
**Jacquet-Smailovic M. [31]**	2022	Anxiety and depression, personality traits, emotional and/or cognitive reactions, quality of life, social support, and potentially stressful events to which the individual may have been exposed	Survivors of major cardiac events, such as MI, are at a significant risk of developing PTSD or PTSS	Different score cutoffs used for probable disorder diagnoses among studies that used the same assessment instrument; the different time periods
**Khan Z. [26]**	2021	The various risk factors and the role of cardiac rehabilitation in reducing the risk of depression in patients after AMI	Depression is common in patients post-ACS and remains undertreated, which can result in higher morbidity and mortality and lead to increased hospital readmission	Due to the lack of uniformity in the types of questionnaires used to collect data in previous studies; not enough data for patient demographics and depression
**Tully P.J. [47]**	2021	The effects of psychological and pharmacological interventions for depression in CAD patients with comorbid depression	Psychological treatments compared to controls and AD compared to placebo, may result in a reduction in depression symptoms at the end of treatment	Single trials lack statistical power, and meta- analyses are limited by the heterogeneous methodological standards of primary studies
**Hannoodee H. [28]**	2021	The impact of schizophrenia on mortality and morbidity outcomes following an initial event of ACS	Higher death rates following ACS in patients who were previously diagnosed with schizophrenia when compared to mentally healthy patients. Greater risk of major complications in schizophrenia patients suffering from ACS after hospital discharge compared to the general population	Retrospective studies, limited by missing data, poor coding, or poor follow-up of participants
**Sancassiani** **F. [29]**	2021	Psychological factors associated with PHD or PDD in people with an ongoing AMI.	Wrong appraisal, interpretation and causal beliefs about symptoms, denial of the severity of the symptoms, and high levels of alexithymia were found related to longer PHD or PDD	Heterogeneity of methods and measures
**Cojocariu S.A. [30]**	2021	The effectiveness of psychological and educational interventions (as an isolated measure or in a cardiac recovery program) compared to the usual care exclusively for patients with acute coronary syndromes	Patients with ACS can receive significant benefits through individualized psychoeducation sessions	A defective distribution between the two genders; the comparison with placebo not applied to psychological and educational interventions, and in all trials the control group was the usual care one; not investigated intervention for other emotional disorders such as BD
**Jacquet-Smailovic M. [25]**	2021	The association between PTSD and IHD	The occurrence of an acute cardiac event is likely to contribute to the development of PTSD	The multiplicity and the heterogeneity of evaluation tools and samples examined. The different time periods studied
**Fernandes N.** **[32]**	2021	The effects of SSRIs on cardiovascular events in depressed CAD patients	The use of SSRIs in post-ACS patients with depression was associated with a 44% relative risk reduction in MI. No difference in mortality	Risk of bias, short follow-up, clinical heterogeneity, absence of subgroup analysis
**Sweda R. [33]**	2020	The effect of AD therapy on mortality and cardiovascular outcomes in patients with ACS	AD in patients following ACS have no effect on mortality but reduce repeat hospitalizations; in patients with depression, there is a reduced risk of recurrent MI with AD therapy	Heterogeneity; insufficient data to identify potentially relevant subgroups; the cumulative number of participants and events was low; patients recruited in RCTs are well selected and might not represent the general population
**Park K. [34]**	2020	The association between duloxetine and CAEs	Duloxetine increased heart rate by 2.22 beats/min and diastolic blood pressure by 0.82 mmHg	Most RCTs were conducted for <13 weeks and each study group’s sample size was <350
**Shao M. [35]**	2020	The revascularization rate in schizophrenic patients after AMI	Patients with schizophrenia and AMI have a lower rate of coronary revascularization as compared with patients without schizophrenia	Limited number of the included studies and their heterogeneity; all retrospective studies
**Haerizadeh M. [36]**	2020	The optimal treatment of PTSD symptoms after medical events such as MI and cancer diagnosis	CBT and EMDR may be promising approaches to reducing PTSD symptoms due to medical event	Few RCTs on this topic; small sample sizes; PTSD symptoms frequently assessed by self-report questionnaires; unblinded patients, substantial heterogeneity
**Papola D. [37]**	2020	The risk of hip fracture, thromboembolism, stroke, MI, pneumonia, and sudden cardiac death associated with exposure to AP	The risk of pneumonia, followed by the risk of hip fracture and thromboembolism, are associated with exposure to AP	The observational nature of the primary studies; significant heterogeneity in terms of populations included in the primary studies; no reanalyzed data by AP class or by individual drug
**Zivkovic S. [38]**	2019	Associations between AP drug use and stroke or MI risk	AP drug use may be associated with an increased risk of stroke, but there is no clear evidence that this risk is further elevated in patients with dementia	Shortcomings of individual studies; confounders rarely adequately adjusted for; the definition of stroke varied considerably across studies; heterogeneity in the definition of AP drug use and duration of follow-up varied widely, from just weeks to 13 years
**Zheng X. [39]**	2019	The efficacy of exercise-based CR treatments in terms of relief from symptoms of anxiety and depression symptoms among patients with MI	For patients with MI, exercise-based CR has been demonstrated to alleviate anxiety and depressive symptoms	A poor level of reporting within the available studies; their study incorporated nine non-RCTs; heterogeneity in the subgroup analysis of anxiety
**Benjenk I. [40]**	2018	Interventions specifically designed to assess or treat mental health symptoms can effectively reduce risk of readmission following hospitalization for physical health conditions	The use of mental health interventions after discharge may be a mechanism for reducing physical health condition readmissions	Suboptimal study designs and small sample sizes; great variation in the readmission outcome measures used by the different studies
**Ladwig S. [41]**	2018	Treatment rates and the application of guidelines in stroke and MI	Despite the high frequency of depression after stroke and MI and the existence of efficacious treatment strategies, people often remain untreated	Variability of the assessment tools used; the reported use of AD may not indicate adequate treatment of depression
**Eurelings L.S. [42]**	2018	Apathy and depressive symptoms in older people are associated with future CVD, stroke, and mortality	Apathy symptoms, irrespective of concurrent depressive symptoms, were associated with a higher risk of MI, stroke, and all-cause CV and non-CV mortality. Depressive symptoms were related to a similarly increased risk of stroke and mortality outcomes, but not of MI	Apathy and depression subscales not validated against a clinical diagnosis; not adjusted all potential confounders; no longitudinal data regarding the development of apathy and depressive symptoms over time
**Richards S.H. [43]**	2018	Mortality, cardiovascular morbidity, and psychological outcomes	Psychological intervention improved psychological symptoms and reduced cardiac mortality for people with CHD	Low ability to judge risk of bias; majority of participants were men post-MI, so poorly generalizable; clinical heterogeneity
**Richards S.H. [44]**	2017	To compare psychological interventions to usual care for coronary heart disease patients, focusing on outcomes like mortality, cardiac morbidity, and psychological well-being, identifying predictors of the effectiveness	Psychological treatments for CHD patients reduced cardiac mortality and relieved psychological symptoms, but no effect on total mortality or risk of revascularization or non-fatal MI	Poor methods, small trials, and short follow up, lack of reporting of study interventions
**Yu Z.H. [45]**	2016	Assess the risk of MI among users of AP	AP use is significantly associated with MI risk, especially among patients with schizophrenia or with drug use during the first 30 days	Potential publication bias, a language bias, inflated estimates by a flawed methodologic design in smaller studies and/or a lack of publication of small trials with opposite results
**Ski C.F. [46]**	2016	Depressive symptoms, mortality (all-cause and cardiac), MI, revascularization, anxiety, social support, and quality of life	Psychosocial interventions, compared with usual care, appear to be effective in reducing depressive symptoms in patients with CHD and depression	Small number of studies included heterogeneity in outcomes and in differences in treatment
**Tully P.J. [27]**	2015	The association between PD, related syndromes, and incident CHD	PD was independently associated with incident CHD, MI, and MACE	Unclear or unblinded determination of CHD outcome, the absence of clear inclusion criteria, retrospective design
**Doyle F. [48]**	2015	Whether post-MI indices could account for found differences in depression	Prevalence of depression post-MI was higher in women, but the association between depression and cardiac prognosis was worse for men	Various heterogeneities; endpoint assessments may pose challenges, but the random intercept addresses study variations
**Janzon E. [49]**	2015	To investigate if physical activity can reduce depression in people with cardiovascular events	Exercise could be effective to reduce the level of depression among CHD patients	Few relevant systematic reviews; old studies from diverse countries and limited evidence
**Jackson J.W. [50]**	2014	Comparing mortality and medical event risk between FGAs and SGAs in older adults; quantify how much medical events explain the observed mortality difference between FGAs and SGAs	Elderly people using FGAs were at higher risk for stroke, ventricular arrhythmia, MI, and hip fracture as compared to SGAs	Individual studies suffered from residual or unmeasured confounding by risk factors for mortality or for the medical event studied
**Prieto M.L. [51]**	2014	Review all available evidence; analyze if people with BD are at higher risk of heart attack or stroke; suggest future research on epidemiology and biomarkers	People with BD have higher risk of stroke and elevated mortality rate, not completely explained by an increased risk of MI	Small number of studies, significant heterogeneity, and dissimilar methodological features
**Whalley B. [52]**	2014	To estimate effects of psychological interventions on cardiac diseases mortality	Psychological interventions did not show strong evidence on reducing total deaths, risk of revascularization, or non-fatal infarction. They did slightly improve depression and anxiety, with a small effect on cardiac mortality	Poor quality of reporting data; substantial heterogeneity; risk of bias
**Bradt J. [53]**	2013	Psychological distress, anxiety, state anxiety, heart and respiratory rate, systolic blood pressure, and pain	Listening to music may have a positive effect on CHD people by reducing blood pressure, heart rate, anxiety, and potentially pain and respiratory rate	Poor quality of reporting, mostly small trials, and high risk of bias
**Health Quality Ontario [54]**	2013	Review effectiveness of depression and/or anxiety screening in adults with chronic diseases	No evidence that screening and treating depression in adults with chronic diseases improved chronic disease outcomes	Heterogeneity in duration of treatments, follow-ups, and different forms of depression
**Foxwell R. [55]**	2013	To examine the connection between illness perceptions, QoL, and mood in a heterogeneous sample of CHD patients	Illness perceptions affect outcomes and disease progression in CHD populations, but no specific model is supported by the results	The combined quality control checklist lacks psychometric testing. Most studies did not state subject representativeness, limiting generalization of findings
**Thombs B.D.** **[56]**	2013	To review depression screening in CHD patients, focusing on screening tool accuracy, treatment effectiveness, and screening’s impact on depression outcomes	Treating depression shows slight symptom improvement in post-MI and stable CHD patients, but not in heart failure patients. Routine depression screening has not proven to improve depression or cardiac outcomes	Substantial heterogeneity; no eligible studies
**Westover A.N. [57]**	2012	Association between prescription stimulant use and adverse cardiovascular outcomes	Most studies in children found no association between stimulant use and adverse cardiovascular outcomes, while that association was more present in adults	Substantial heterogeneity; risk of bias
**Prochaska J.J. [58]**	2012	To examine the risk of treatment emergent, cardiovascular serious adverse events associated with varenicline use for tobacco cessation	No significant increase in cardiovascular serious adverse events associated with varenicline use	Bias in methods
**Whalley B. [59]**	2011	Determine the independent effects of psychological interventions in patients with CHD and explore study-level predictors of the impact of these interventions	Psychological treatments appear effective in treating psychological symptoms of CHD patients	The lack of methodological detail; substantial heterogeneity for psychological outcomes; risk of bias
**Zuidersma M. [60]**	2011	Evaluate if depressed ACS patients face different risks based on recurrence and timing of depressive episodes	There is no consistent evidence to prove that ACS patients with first and new onset depression are at particularly risk of worse prognosis	Few included studies; substantial heterogeneity
**Thombs B.D. [61]**	2008	Evaluation of the potential benefits of depression screening in patients with CVD	Depression treatment in people with CVDs improves depression symptoms but does not affect cardiac outcomes	Heterogeneity in outcomes and in differences in treatment. Not enough evidence to assess potential harms related to screening or treatment
**Thombs B.D. [62]**	2007	To assess performance characteristics of depression screening instruments after acute MI	Depression treatment in people with CVDs improves depression symptoms but does not affect cardiac outcomes	Small samples, low quality, and limited information. Inconsistencies in results related to diagnostic usefulness
**Van der Kooy K. [63]**	2007	To estimate the risk of depression as an independent risk factor for various CVD and explore the effects of heterogeneity and methodological quality	Depression seems to be an independent risk factor for the onset of a wide range of CVDs, although this evidence is related to a high level of heterogeneity	Missing some studies, for instance because of non-journal publication; substantial heterogeneity
**Swenson J.R. [64]**	2006	To examine whether SSRIs were associated with an increased or decreased risk of CAEs	Review on AD in high-risk patients did not determine if SSRIs increase or decrease cardiovascular risks	Rarity of serious AEs, lack of large trials and lack of adequate reporting of AEs in published trials
**Frasure-Smith N. [65]**	2005	To officially recognize depression as a cardiac risk factor	Data recognizes depression as a risk factor for both the development and worsening of CHD	Multiple methodological differences in RCTs
**Sørensenf C. [66]**	2005	To assess, in patients with MI, the methodological quality and to test whether depression leads to an increased post-MI mortality	No conclusion	Low number of studies of acceptable methodological quality; mixed results; risk of bias
**Rees K. [67]**	2004	To determine the effectiveness of psychological interventions on mortality and morbidity, psychological measures, QoL, and modifiable cardiac risk factors in patients with CHD	Overall psychological interventions showed no effect on total or cardiac mortality but did show small reductions in anxiety and depression in patients with CHD	Poor quality of trials, considerable heterogeneity, and risk of publication bias

AD: antidepressants; RCTs: randomized controlled trials; MI: myocardial infarction; CHD: coronary heart disease; CVDs: cardiovascular diseases; ADHD: attention deficit hyperactivity disorder; SMI: severe mental illness; ACS: acute coronary syndromes; BD: bipolar disorder; MACEs: major adverse cardiac events; AMI: acute myocardial infarction; CAD: coronary artery disease; PTSD: post-traumatic stress disorder; PTSS: potentially stressful events; PHD: pre-hospital delay; PDD: patients’ decisional delay; IHD: ischemic heart disease; SSRIs: selective serotonin reuptake inhibitors; CAEs: coronary adverse events; CBT: cognitive behavioral therapy; EMDR: eye movement desensitization and reprocessing; AP: antipsychotics; CR: cardiac rehabilitation; CV: cardiovascular; PD: panic disorder; FGAs: first-generation antipsychotics; SGAs: second-generation antipsychotics; QoL: quality of life; AEs: adverse events.

## 4. Discussion

The brain–heart axis is an intra- and bidirectional connection between the central nervous system and cardiovascular system. The relationship between psychiatric disease and cardiovascular disease has been a topic of great interest in the medical community for many years. It is well established that individuals with psychiatric disorders, such as depression, anxiety, and bipolar disorder, are at increased risk of developing cardiovascular disease. This relationship is complex and multifaceted, involving a combination of biological, psychological, and social factors. Understanding the impact of psychiatric disease on the incidence, management, and outlook of cardiovascular disease is critical for improving patient outcomes and reducing healthcare costs.

The underlying mechanisms behind this association are not yet fully understood, but several theories have been proposed. One possible explanation is that psychiatric disorders and cardiovascular disease share common risk factors, such as smoking, sedentary lifestyle, and poor diet. Additionally, chronic stress and inflammation, which are often present in psychiatric disorders, have been linked to the development of cardiovascular disease. The presence of a psychiatric disorder can complicate the management of cardiovascular disease in several ways. For example, individuals with depression may be less likely to adhere to treatment regimens, engage in healthy behaviors, and follow up with healthcare providers. This can result in the suboptimal control of cardiovascular risk factors, such as high blood pressure and high cholesterol, leading to worsened outcomes. The detection and management of risk factors for cardiovascular disease in psychiatric patients result crucial for prevention.

This umbrella review contributes to demonstrating that the existing link between psychiatric disorders and CVDs deserves considerable attention [68]. The increased risk of cardiovascular disease associated with mental illness can be considered multifactorial and can be attributed to several and complex pathways, with the possible implication of biological, behavioral, psychological, and genetic mechanisms [2]. Our findings confirm the frequent comorbidity between psychiatric disorders such as depression, anxiety, schizophrenia and CVDs, with consequential increased risk of major adverse events, complications, and mortality. There is also evidence that depression, anxiety, and post-traumatic stress disorder (PTSD) can develop after cardiac events.

Mental health treatment has as a significant impact on outcomes in patients with CVDs, in terms of reduced hospitalizations, emergency department visits, and even improved survival [69,70], although there is some evidence for potential cardiovascular risks associated with psychotropic drugs, particularly antipsychotics (cardiac arrhythmias, tachycardia, and QT interval prolongation). Recent studies corroborate the attitude that selective-serotonin reuptake inhibitors (SSRIs) are well-tolerated agents, but research regarding the association between SSRIs and cardiovascular adverse events is still controversial [71,72]. These data suggest that clinicians should carefully consider the cardiovascular risk of psychotropic drugs, choose the appropriate type and dose of psychotropic drugs in patients with CVDs, and should constantly monitor the progress of treatment.

Psychosocial stressors, emotional and cognitive reactions to life events, and the overall quality of life are key factors in determining the cardiovascular health of individuals suffering from psychiatric disorders. Patients with mental health disorders are at greater risk of adopting behaviors such as smoking, inactive lifestyle, or failure to take prescribed medications, thus increasing the probability of experiencing a heart disease event. Unhealthy behaviors, social stress, and poor social support contribute both to worsening the progress of psychiatric diseases and to increasing the morbidity and mortality rates in patients with heart disease [73], creating a vitious circle that is detrimental to psychiatric and cardiologic outcomes and response to treatments.

**Figure 1:**
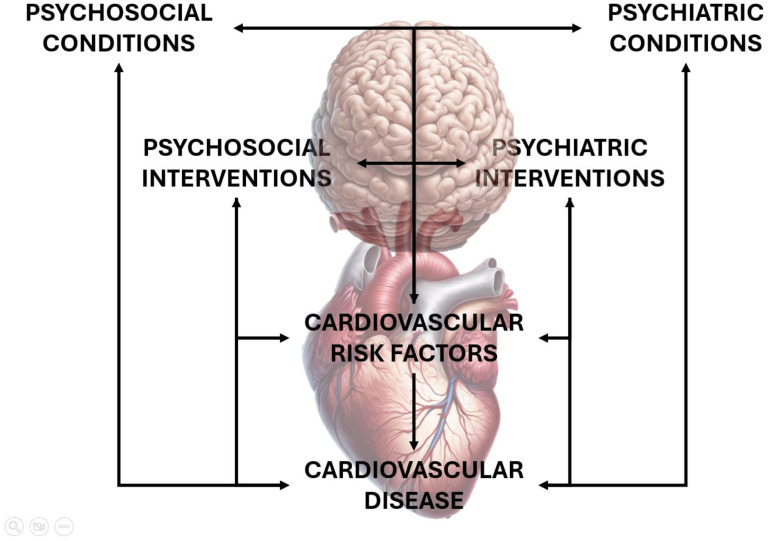
Interplay between psychosocial, psychiatric, and cardiovascular conditions as well as treatments.

Heart disorders greatly alarm, even unconsciously, as the heart is an organ heavily invested with symbolic meanings. It is considered the guardian of emotions and, through its regular rhythm, gives a sense of health instilling the certainty of being alive. Many physical symptoms caused by anxiety can lead to even more anxiety, as they mimic other serious health issues and can be very alarming. For instance, experiencing sudden, unexpected tachycardia with no apparent cause can be terrifying. The rapid heartbeat is a common symptom of both panic attacks and tachycardia, and if there is no awareness that one has a “frozen” state of anxiety, the lack of a clear trigger for the attack can be very frightening. Every cardiac disorder causes concern, but this does not necessarily prompt individuals to obtain a check-up. In fact, due to a mechanism of avoidance and denial, not seeking a check-up wrongly leads to the belief that there is nothing wrong. This kind of unconscious dynamic exposes individuals to serious risks, as it results in neglecting preventive measures, avoiding necessary check-ups, and inaccurately following prescribed therapies or even forgetting to take them altogether. Unintentional forgetfulness, “missed acts” or “lapses of action”, occur when one intends to take a certain action but instead does another. In the case of medications, this can happen because taking them means becoming aware of having an illness and getting in touch with the distress this causes. In fact, they can be considered depressive equivalents, i.e., depressive symptoms in the form of “forgetfulness” [74]. In such perspective, psychosocial interventions, psychological therapies, and cardiac rehabilitation assume a pivotal role to improve compliance to pharmacological treatments, to promote health-related quality of life, to increase healthy behaviors, and to enhance resilience and self-efficacy (Figure 1). The psychological treatment helps to become aware of the even unconscious functioning that gives rise to mental illness. It allows individuals to observe their lifestyle, how they work, live, and interact socially. Additionally, it activates reflective capacity over time, enabling individuals to make sense of their lives and place them in the context of personal history. This intervention also helps restore the mind–body relationship that is crucial for maintaining or regaining good inner balance and physical well-being throughout life.

One of the key strengths of this umbrella review is a comprehensive literature search and rigorous screening process for selecting studies. Despite this, there are also some limitations that must be taken into account. One limitation refers to publication bias, as studies reporting statistically significant findings are more likely to be published, potentially impacting the evidence synthesis. Moreover, a further limitation is the uncertainty surrounding causality and directionality: while significant associations have been found between psychiatric disorders and cardiovascular disease in many studies, the exact causal mechanisms and directional influences are not fully understood. The studies included patients from a variety of demographics, with differences in age, gender, and clinical features, which could possibly complicate the analysis of the results. The review encompasses studies employing different designs and methodologies, potentially limiting the generalizability of the findings. Future studies should focus on overcoming the limitations aforementioned by employing longitudinal designs, integrating multiple approaches to investigate comprehensive mechanisms, and exploring different ways to cope with psychiatric disorders across different stages. Furthermore, translational studies are crucial for converting research findings into personalized interventions and therapeutic approaches for individuals affected by psychiatric disorders in comorbidity with cardiovascular disease.

The intricate connection between the brain and heart creates a dynamic relationship that can impact overall health. The impact of psychiatric disease on the incidence, management, and outlook of cardiovascular disease is a significant clinical and public health concern. Individuals with psychiatric disorders are at increased risk of developing cardiovascular disease, which can lead to worse outcomes and increased healthcare costs. Addressing this issue requires a coordinated effort between mental health and cardiovascular specialists, as well as a focus on prevention, early detection, and integrated care. It is crucial for healthcare providers to recognize and address the complex interplay between psychiatric disorders and cardiovascular diseases in order to improve outcomes for this vulnerable population [75].

This work has several limitations. First, while we considered adding other search engines for our bibliographic search, we preferred to limit this to PubMed to maximize yield and efficiency, while recognizing that any review of at least adequate quality would have been indexed there. Yet, included reviews searched many databases, including CENTRAL, CINAHL, Embase, Scopus, PsychInfo, and Web of Science, thus providing a veritable guarantee of comprehensiveness. Given the design of our work (i.e., an umbrella review), individual socio-demographic details could not be appropriately summarized, and the attentive reader should instead direct their attention to the primary studies pooled by the reviews we have included. In addition, it is important to acknowledge that recent or current research on the intersection of psychiatric and cardiovascular health appeared often limited by methodological challenges such as heterogeneity in study designs, small sample sizes, and varied assessment tools. These issues highlight the need for more rigorous, well-designed studies to provide clearer insights and more reliable data to inform clinical practices and health policies effectively. Indeed, our work, while comprehensive, leaves ample room for additional studies and evidence synthesis efforts, including a much-needed patient-level meta-analysis.

This umbrella review provides a comprehensive overview of the current evidence on the relationship between psychiatric disease and cardiovascular disease, highlighting the need for further research and interventions in this area. By better understanding the mechanisms underlying this association and implementing targeted strategies to improve outcomes, we can help reduce the burden of cardiovascular disease in individuals suffering from psychiatric disorders.

Given the pressing necessity to appropriately manage patients with mental illnesses and CVDs, by providing more tailored and effective interventions, further accurate research is warranted. Moreover, a careful and informed collaboration between cardiovascular experts and mental health professionals should be established to advance and to refine the care of patients suffering from psychiatric diseases and CVDs.

## Data Availability

The data presented in this study are available on request from the corresponding author.

## Supplementary Materials

The following supporting information can be downloaded at: https://www.mdpi.com/article/10.3390/life14080919/s1, Table S1: Appraisal of the quality of included reviews according to the Oxman and Guyatt index.
