# Supplementary material for: The Brain–Heart Axis: An Umbrella Review on Impact of Psychiatric Disease on Incidence, Management, and Outlook of Cardiovascular Disease
Source: Life (Basel). 2024 Jul 23;14(8):919. doi: 10.3390/life14080919 (PMC11355298; doi:10.3390/life14080919)
Supplement: Supplementary file 1 [file life-14-00919-s001.zip › life-3066285-supplementary.pdf]

Online Supplement

Table S1. Appraisal of the quality of included reviews according to the Oxman and Guyatt index.

First author	Year	Explicit search methods	Comprehensive search	Explicit selection methods	Bias in selection minimized	Explicit validity criteria	Validity assessed	Explicit pooling methods	Bias in pooling minimized	Conclusion supported by data	Overall quality score (from 1 - extensively flawed to 7 - minimally flawed)
**Kőhler-Forsberg O.**	2023	Yes	Yes	Yes	Yes	Yes	Yes	Yes	Yes	Yes	7
**Zhu C.**	2023	Yes	Yes	Yes	Yes	Yes	Yes	Yes	Yes	Yes	7
**Chen Y.**	2023	Yes	Yes	Yes	Yes	Yes	Yes	Yes	Yes	Yes	7
**Aw P.Y.**	2023	Yes	Yes	Yes	Yes	Yes	Yes	Yes	Yes	Yes	7
**Zhang L.**	2022	Yes	Yes	Yes	Yes	Yes	Yes	Yes	Yes	Yes	7
**Chan J.K.N.**	2022	Yes	Yes	Yes	Yes	Yes	Yes	Yes	Yes	Yes	7
**Khan Z.**	2021	Yes	Yes	Yes	Yes	Yes	Yes	Yes	Yes	Yes	7
**Tully P.J.**	2021	Yes	Yes	Yes	Yes	Yes	Yes	Yes	Yes	Yes	7
**Jacquet-Smailovic M.**	2022	Yes	Yes	Yes	Yes	Yes	Yes	Yes	Yes	Yes	7
**Hannoodee H.**	2021	Yes	Yes	Yes	Yes	Yes	Yes	Yes	Yes	Yes	7
**Sancassiani F.**	2021	Yes	Yes	Yes	Yes	Yes	Yes	Yes	Yes	Yes	7
**Cojocariu S.A.**	2021	Yes	Yes	Yes	Yes	Yes	Yes	Yes	Yes	Yes	7
**Jacquet-Smailovic M.**	2021	Yes	Yes	Yes	Yes	Yes	Yes	Yes	Yes	Yes	7
**Sweda R.**	2020	Yes	Yes	Yes	Yes	Yes	Yes	Yes	Yes	Yes	7
**Fernandes N.**	2021	Yes	Yes	Yes	Yes	Yes	Yes	Yes	Yes	Yes	7
**Park K.**	2020	Yes	Yes	Yes	Yes	Yes	Yes	Yes	Yes	Yes	7

**Shao M.**	2020	Yes	Yes	Yes	Yes	Yes	Yes	Yes	Yes	Yes	7
**Haerizadeh M.**	2020	Yes	Yes	Yes	Yes	Yes	Yes	Yes	Yes	Yes	7
**Papola D.**	2020	Yes	Yes	Yes	Yes	Yes	Yes	Yes	Yes	Yes	7
**Zivkovic S.**	2019	Yes	Yes	Yes	Yes	Yes	Yes	Yes	Yes	Yes	7
**Zheng X.**	2019	Yes	Yes	Yes	Yes	Yes	Yes	Yes	Yes	Yes	7
**Benjenk I.**	2018	Yes	Yes	Yes	Yes	Yes	Yes	Yes	Yes	Yes	7
**Ladwig S.**	2018	Yes	Yes	Yes	Yes	Yes	Yes	Yes	Yes	Yes	7
**Eurelings L.S.**	2018	Yes	Yes	Yes	Yes	Yes	Yes	Yes	Yes	Yes	7
**Richards S.H.**	2018	Yes	Yes	Yes	Yes	Yes	Yes	Yes	Yes	Yes	7
**Richards S.H.**	2017	Yes	Yes	Yes	Yes	Yes	Yes	Yes	Yes	Yes	7
**Yu Z.H.**	2016	Yes	Yes	Yes	Yes	Yes	Yes	Yes	Yes	Yes	6
**Ski C.F.**	2016	Yes	Yes	Yes	Yes	Yes	Yes	Yes	Yes	Yes	7
**Tully P.J.**	2015	Yes	Yes	Yes	Yes	Yes	Yes	Yes	Yes	Yes	7
**Doyle F.**	2015	Yes	Yes	Yes	Yes	Yes	Yes	Yes	Yes	Yes	7
**Janzon E.**	2015	Yes	Yes	Yes	Yes	Yes	Yes	Yes	Yes	Yes	6
**Jackson J.W.**	2014	Yes	Yes	Yes	Yes	Yes	Yes	Yes	Yes	Yes	6
**Prieto M.L.**	2014	Yes	Yes	Yes	Yes	Yes	Yes	Yes	Yes	Yes	6
**Bradt J.**	2013	Yes	Yes	Yes	Yes	Yes	Yes	Yes	Yes	Yes	6

**Health Quality Ontario**	2013	Yes	Yes	Yes	Yes	Yes	Yes	Yes	Yes	Yes	6
**Foxwell R .**	2013	Yes	Yes	Yes	Yes	Yes	Yes	Yes	Yes	Yes	6
**Thombs B.D.**	2013	Yes	Yes	Yes	Yes	Yes	Yes	Yes	Yes	Yes	6
**Whalley B.**	2014	Yes	Yes	Yes	Yes	Yes	Yes	Yes	Yes	Yes	6
**Westover A.N.**	2012	Yes	Yes	Yes	Yes	Yes	Yes	Yes	Yes	Yes	6
**Prochaska J.J.**	2012	Yes	Yes	Yes	Yes	Yes	Yes	Yes	Yes	Yes	6
**Whalley B.**	2011	Yes	Yes	Yes	Yes	Yes	Yes	Yes	Yes	Yes	6
**Zuidersma M.**	2011	Yes	Yes	Yes	Yes	Yes	Yes	Yes	Yes	Yes	5
**Thombs B.D.**	2008	Yes	Yes	Yes	Yes	Yes	Yes	Yes	Yes	Yes	5
**Thombs B.D.**	2007	Yes	Yes	Yes	Yes	Yes	Yes	Yes	Yes	Yes	6
**Swenson J.R.**	2006	Yes	Yes	Yes	Yes	Yes	Yes	Yes	Yes	Yes	6
**Van der Kooy K.**	2007	Yes	Yes	Yes	Yes	Yes	Yes	Yes	Yes	Yes	5
**Frasure-Smith N.**	2005	Yes	Yes	Yes	No	Yes	Yes	Yes	No?	Yes	4
**Sørensenf C.**	2005	Yes	Yes	Yes	No	Yes	Yes	Yes	No?	Yes?	4
**Rees K.**	2004	Yes	Yes	Yes	Yes	Yes	Yes	Yes	Yes	Yes	5

The Oxman and Guyatt index is a tool used for assessing the quality of systematic reviews. It helps determine the reliability and validity of the findings presented in such reviews. The index consists of these criteria, each addressing different aspects of the review's methodology and reporting. Here is a detailed list of the criteria along with explanations:

1. Explicit search methods: this criterion evaluates whether the systematic review clearly describes the search methods used to identify studies. Explicit search methods include specifying the databases searched, the search terms used, and the time period covered. This transparency ensures that the search process can be replicated and assessed for comprehensiveness.
2. Comprehensive search: this assesses if the review conducted a thorough search for relevant studies, covering multiple databases, gray literature, and other sources. A comprehensive search increases the likelihood of capturing all relevant studies and minimizes the risk of publication bias.
3. Explicit selection methods: this criterion examines whether the review explicitly states the criteria and process for selecting studies for inclusion. Clear selection methods ensure that the selection process is systematic, unbiased, and reproducible.
4. Bias in selection minimized: this evaluates whether the review took steps to minimize bias during the study selection process. This may include using multiple reviewers to independently screen studies and resolve disagreements through discussion or a third party. Minimizing selection bias helps in ensuring the included studies are representative of the available evidence.
5. Explicit validity criteria: this criterion assesses whether the review clearly defines the criteria for evaluating the validity (quality) of the included studies. Explicit validity criteria

help in systematically assessing the methodological quality of the studies.
6. Validity assessed: this examines if the review actually applied the predefined validity criteria to assess the included studies. Assessing validity helps in determining the reliability and risk of bias in the study findings.
7. Explicit pooling methods: this criterion looks at whether the review clearly describes the methods used for pooling data from individual studies, such as meta-analysis techniques. Explicit pooling methods ensure that the data synthesis process is transparent and appropriate.
8. Bias in pooling minimized: this evaluates whether the review took steps to minimize bias in the pooling process. This may include assessing heterogeneity, using appropriate statistical methods, and conducting sensitivity analyses. Minimizing bias in pooling ensures that the synthesized results are reliable and valid.
9. Conclusion supported by data: this criterion assesses whether the conclusions drawn by the review are justified by the data presented. Conclusions should be based on a comprehensive and unbiased synthesis of the evidence, ensuring they are reliable and applicable.
Each item was scored with a Yes or a No, and a comprehensive score was given using 1 and 0 weights for Yes and No respectively.
